# Characterization of Amino Acid Substitutions in the Two-Component Regulatory System AdeRS Identified in Multidrug-Resistant Acinetobacter baumannii

**DOI:** 10.1128/msphere.00709-21

**Published:** 2021-11-24

**Authors:** K. Lucaßen, K. Xanthopoulou, J. Wille, T. Wille, Y. Wen, X. Hua, H. Seifert, P. G. Higgins

**Affiliations:** a Institute for Medical Microbiology, Immunology and Hygiene, Faculty of Medicine and University Hospital Cologne, University of Cologne, Cologne, Germany; b German Center for Infection Research (DZIF), Partner Site Bonn-Cologne, Cologne, Germany; c Talent Highland and Center for Gut Microbiome Research of Med-X Institute, The First Affiliated Hospital, Xi'an Jiaotong University, Xi’an, China; d Department of Infectious Diseases, Sir Run Run Shaw Hospital, Zhejiang University School of Medicine, Hangzhou, China; e Key Laboratory of Microbial Technology and Bioinformatics of Zhejiang Province, Hangzhou, China; f Regional Medical Center for National Institute of Respiratory Diseases, Sir Run Run Shaw Hospital, School of Medicine, Zhejiang University, Hangzhou, China; Antimicrobial Development Specialists, LLC

**Keywords:** efflux pump, AdeABC, tigecycline

## Abstract

In Acinetobacter baumannii, resistance-nodulation-cell division (RND)-type efflux is a resistance mechanism of great importance since it contributes to reduced susceptibility to multiple antimicrobial compounds. Some mutations within the genes encoding the two-component regulatory system AdeRS appear to play a major role in increased expression of the RND efflux pump AdeABC and, consequently, in reduced antimicrobial susceptibility, as they are commonly observed in multidrug-resistant (MDR) A. baumannii. In the present study, the impact of frequently identified amino acid substitutions, namely, D21V and D26N in AdeR and T156M in AdeS, on *adeB* expression, efflux activity, and antimicrobial susceptibility was investigated. Reverse transcription-quantitative PCR (qRT-PCR) studies revealed significantly increased *adeB* expression caused by D26N (AdeR) and T156M (AdeS). In addition, accumulation assays have shown that these mutations induce increased efflux activity. Subsequently, antimicrobial susceptibility testing via agar dilution and broth microdilution confirmed the importance of these substitutions for the MDR phenotype, as the MICs for various antimicrobials of different classes were increased. In contrast, the amino acid substitution D21V in AdeR did not lead to increased *adeB* expression and did not reduce antimicrobial susceptibility. This study demonstrates the impact of the D26N (AdeR) and T156M (AdeS) amino acid substitutions, highlighting that these regulators represent promising targets for interfering with efflux activity to restore antimicrobial susceptibility.

**IMPORTANCE** The active efflux of antimicrobials by bacteria can lead to antimicrobial resistance and persistence and can affect multiple different classes of antimicrobials. Efflux pumps are tightly regulated, and their overexpression can be mediated by changes in their regulators. Identifying these changes is one step in the direction of resistance prediction, but it also opens the possibility of targeting efflux pump regulation as a strategy to overcome antimicrobial resistance. Here, we have investigated commonly found changes in the regulators of the main efflux pumps in Acinetobacter baumannii.

## INTRODUCTION

Acinetobacter baumannii has emerged as a serious nosocomial pathogen and has been implicated in various hospital outbreaks. It predominantly affects compromised and intensive care unit patients, causing ventilator-associated pneumonia, meningitis, and wound, urinary tract, and bloodstream infections (1).

Multidrug resistance (MDR) is widespread among clinical A. baumannii isolates. In particular, the rise of carbapenem-resistant isolates is of major concern, indicating the need for novel treatment approaches (2). Resistance to the vast majority of antibiotic classes is primarily acquired through horizontal gene transfer, as can be observed from the dissemination of carbapenemase-encoding genes, or based on target-site mutations such as amino acid substitutions within GyrA and ParC, which are responsible for fluoroquinolone resistance (3–5). Both horizontal gene transfer and target site mutations are usually very specific, affecting a limited spectrum of antimicrobial compounds or antimicrobial classes.

In this respect, the above-described mechanisms differ from intrinsic efflux mechanisms, which are represented in particular by the resistance-nodulation-cell division (RND) family. The chromosomally encoded RND-type efflux pumps are tripartite and composed of an inner membrane-located pump and an outer membrane pore, which are connected via a linker protein. The broad range of RND substrates can reduce susceptibility of A. baumannii to multiple antimicrobials of different classes, as well as antiseptics, detergents, heavy metals, and disinfectants (6–11).

Characterized RND efflux pumps in A. baumannii are AdeABC, AdeFGH, and AdeIJK, whose expression is controlled by the two-component systems AdeRS (AdeABC), the LysR-like transcriptional regulator AdeL (AdeFGH), or the TetR-like repressor AdeN (AdeIJK) (12–14). Overexpression of AdeABC or AdeIJK has been shown to cause reduced antimicrobial susceptibility (15, 16). It was shown that overexpression of AdeABC is caused by amino acid substitutions within the dimerization and histidine-containing phosphotransfer domain (DHp) of its regulator sensor kinase AdeS, which includes the phosphorylation residue H149 (17–20). Moreover, amino acid substitutions within the receiver domain of the corresponding response regulator AdeR are also associated with AdeABC overexpression. In particular, changes of residues in spatial proximity of the phosphorylation site D63 have been shown to affect the function of AdeR (21, 22).

During a previous study of clinical southern European A. baumannii isolates, the AdeR double substitution D21V and D26N was observed in 17 of 65 isolates with high tigecycline MICs (18). The same double substitution was identified in a worldwide study; however, it was only found in a single European isolate (17). Within the same tigecycline surveillance study, the AdeS amino acid substitution T156M was identified in six different isolates with high tigecycline MICs from Asia and North and Latin America (17). The aim of this study was to investigate these hitherto-uncharacterized amino acid substitutions in terms of their contribution to increased efflux activity and reduced antimicrobial susceptibility (17, 18).

## RESULTS

### Antimicrobial susceptibility.

The amino acid substitutions D21V and D26N have been identified in multiple clinical isolates in two different studies and were hypothesized to represent resistance mutations (17, 18). To characterize these substitutions, the *adeRS* knockout strain derived from ATCC 17978 was recomplemented with *adeRS* of the A. baumannii reference strain ACICU and cloned into the shuttle vector pJN17/04 with and without the corresponding nucleotide exchanges. To determine the impact of the amino acid substitutions, the mutant strains were subjected to antimicrobial susceptibility testing against eight different antimicrobial classes. MIC results are summarized in Table 1.

**TABLE 1:** MICs determined by agar dilution for amikacin, azithromycin, chloramphenicol, ciprofloxacin, erythromycin, gentamicin, meropenem, minocycline, levofloxacin, rifampin, and tetracycline and by broth microdilution for tigecycline^*a*^

Strain	MIC (mg/liter) of:											
	AMK	GEN	AZI	ERY	CIP	LVX	CHL	MEM	RIF	MIN	TET	TGC
17978 *adeR*-wt	4	**8**	16	16	0.5	0.25	64	1	4	≤0.125	2	0.5
17978 *adeR*-D26N	8	**16**	32	32	1	0.5	64	2	4	0.25	4	2
17978 *adeR*-D21V	4	4	8	16	0.25	0.25	64	0.5	4	≤0.125	2	0.5
17978 *adeR*-D21V+D26N	8	**16**	32	32	1	0.5	64	1	4	0.25	4	2
ACICU	**>128**	**32**	32	32	**>128**	**32**	>128	1	8	2	32	2
ABC153	**>128**	**>128**	>128	>128	**>128**	**32**	>128	**64**	8	32	>128	8
ABC154	**>128**	**>128**	>128	>128	**64**	**16**	>128	**64**	8	16	>128	2

a AMK, amikacin; AZM, azithromycin; CHL, chloramphenicol; CIP, ciprofloxacin; ERY, erythromycin; GEN, gentamicin; MEM, meropenem; MIN, minocycline; LVX, levofloxacin; RIF, rifampin; TET, tetracycline (TET); TGC, tigecycline. Bold indicates resistance determined by EUCAST breakpoints (33).

In comparison to 17978 *adeR*-wt, the amino acid substitution D26N in 17978 *adeR*-D26N caused a reduction in the susceptibility to aminoglycosides, macrolides, fluoroquinolones, carbapenems, and tetracyclines, including tigecycline. On the other hand, changing the amino acid from aspartic acid to valine at residue 21 of AdeR in 17978 *adeR*-D21V had no effect in antimicrobial susceptibility. However, combining D21V with D26N in 17978 *adeR*-D21V+D26N revealed the same antimicrobial resistance phenotype as the single D26N substitution in 17978 *adeR*-D26N, apart from a minor increase of the meropenem MIC. These data suggest that D21V has little or no impact on antimicrobial susceptibility.

Furthermore, the clinical isolate pair A. baumannii ABC153 and ABC154 was analyzed. These two isolates were genetically identical apart from a T156M amino acid substitution in AdeS of ABC153 (17). Within this isolate pair, the most striking change was revealed for tigecycline. The *adeS* wild-type strain ABC154 had a tigecycline MIC of 2 mg/liter, whereas ABC153 revealed a tigecycline MIC of 8 mg/liter. Furthermore, ABC153 revealed higher MICs for fluoroquinolones and minocycline.

### AdeB expression studies.

**FIG 1:**
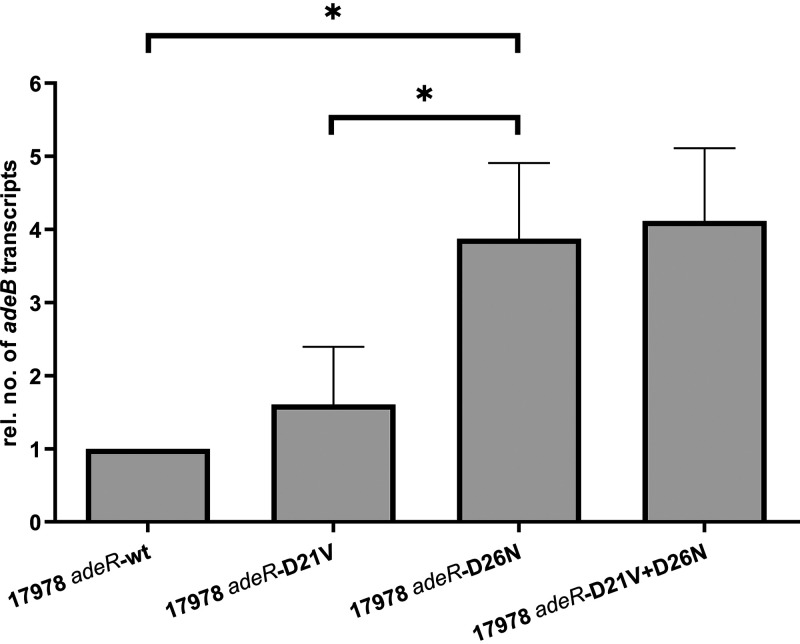
Relative *adeB* expression of A. baumannii ATCC 17978-derived *adeR* mutant strains determined by qRT-PCR. Results are represented as means ± standard errors of the means. Statistical analysis was done by using an unpaired *t* test of the absolute values. *, *P < *0.015.

**FIG 2:**
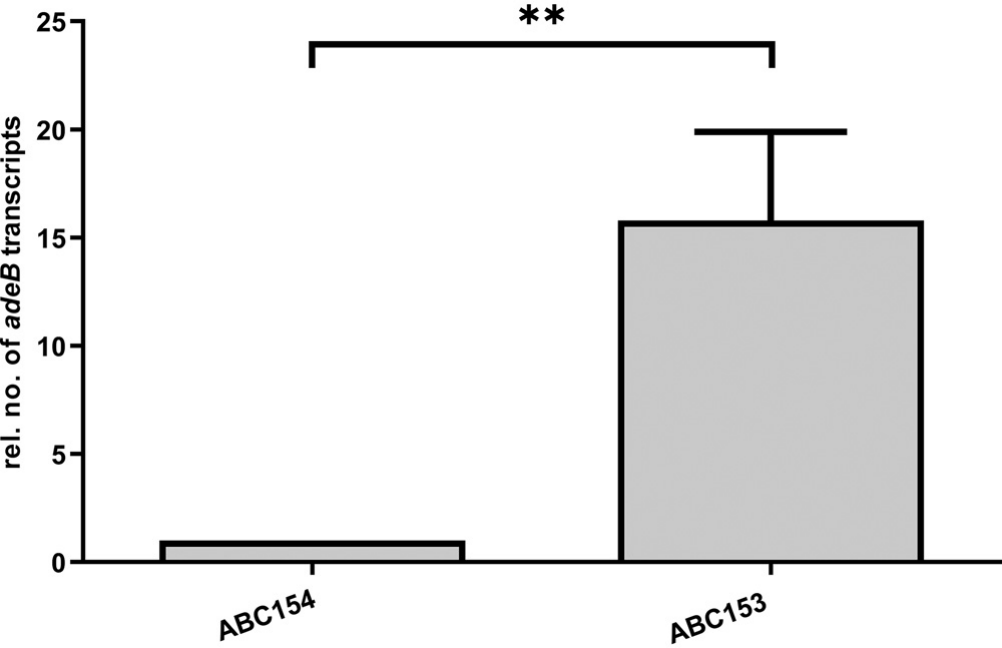
Relative *adeB* expression of A. baumannii isolates ABC153 and ABC154 determined by qRT-PCR. Results are represented as means ± standard errors of the means. Statistical analysis was done by using an unpaired *t* test of the absolute values. **, *P < *0.01.

To determine whether the amino acid substitutions within AdeRS affect *adeB* expression, reverse transcription-quantitative PCR (qRT-PCR) was performed. Figure 1 shows that insertion of the D21V amino acid substitution into the WT *adeRS* backbone did not lead to a significant increase in expression of *adeB* (*P = *0.6876). On the other hand, 17978 *adeR*-D26N exhibited a significant increase in *adeB* expression by a factor of 3.9-fold (*P = *0.0143), compared to 17978 *adeR*-wt. Furthermore, comparing 17978 *adeR*-D21V and 17978 *adeR*-D26N revealed a 2.4-fold higher *adeB* expression for the strain carrying the D26N mutation (*P = *0.0106). Additionally, combining D21V and D26N caused an *adeB* expression of a similar level to D26N alone. Analysis of *adeB* expression in the clinical isolate pair ABC153 and ABC154 exhibited a 15.8-fold higher expression in ABC153 (*P *= 0.0094), which is carrying the T156M amino acid substitution in AdeS, compared to ABC154 (Fig. 2).

### Accumulation assay.

**FIG 3:**
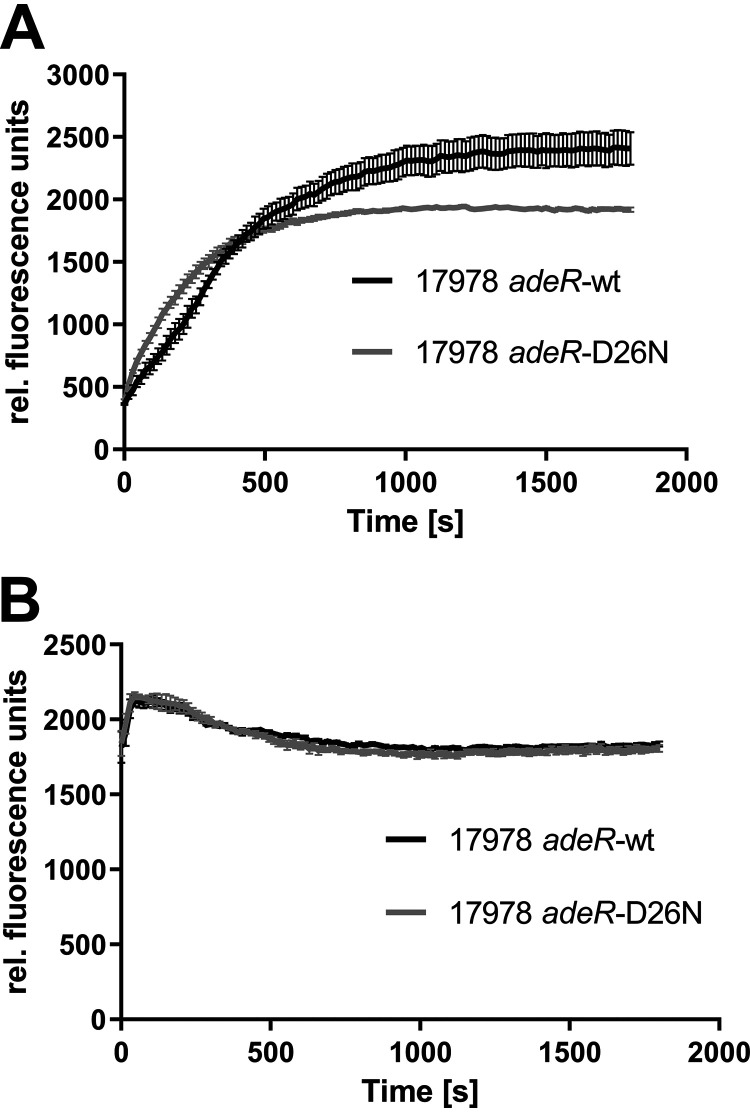
Ethidium bromide (EtBr)accumulation in ATCC 17978 Δ*adeRS* transformants that were untreated (A) and treated with CCCP (500 μm) (B). Fluorescence was measured every 15 s over 30 min. Data were collected from three independent experiments and are presented as means ± standard errors of the means.

**FIG 4:**
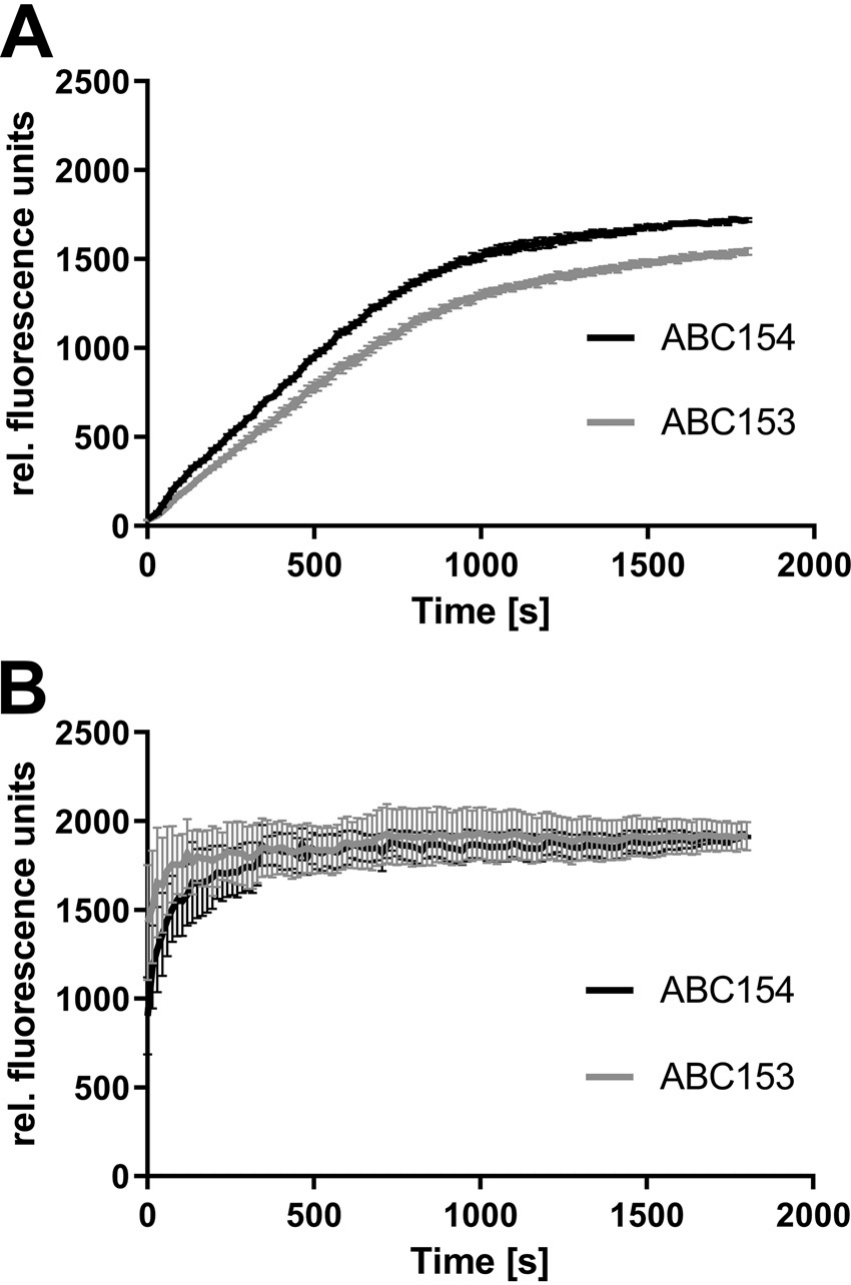
EtBR accumulation of clinical isolates ABC153 and ABC154 that were untreated (A) and treated with CCCP (500 μm) (B). Fluorescence was measured every 15 s over 30 min. Data were collected from three independent experiments and are presented as means ± standard errors of the means.

To verify the direct correlation of increased *adeB* expression and efflux activity, the accumulation of ethidium was measured. Whereas insertion of the AdeR D21V mutation did not cause altered ethidium accumulation levels (Fig. S1 in the supplemental material), 17978 *adeR*-D26N revealed 25% lower ethidium accumulation in comparison to 17978 *adeR*-wt, indicating higher efflux activity induced by the D26N amino acid substitution in AdeR (Fig. 3A). Similar findings were revealed for the clinical isolate pair ABC153 and ABC154. Here, the *adeS* wild-type strain ABC154 was found to exhibit 12% higher accumulation than ABC153 (Fig. 4A). This effect was abolished within the compared strain couples by performing the ethidium accumulation assay in the presence of the proton motive force uncoupler carbonyl cyanide *m*-chlorophenylhydrazone (CCCP). Inhibiting efflux with CCCP caused almost identical levels of ethidium accumulation for 17978 *adeR*-wt and 17978 *adeR*-D26N (Fig. 3B) and also for the isolates ABC153 and ABC154 (Fig. 4B). These results confirm that the described differences of ethidium accumulation are associated with increased efflux activity.

10.1128/msphere.00709-21.2 FIG S1 Comparison of EtBR accumulation between 17978 *adeR*-wt and 17978 *adeR-*D21V. Fluorescence was measured every 15 s over 30 min. Data were collected from three independent experiments and are presented as means ± standard errors of the means. Download FIG S1, TIF file, 0.5 MB. Copyright © 2021 Lucaßen et al. 2021 Lucaßen et al. https://creativecommons.org/licenses/by/4.0/ This content is distributed under the terms of the Creative Commons Attribution 4.0 International license.

## DISCUSSION

The contribution of mutations in the two-component regulatory system encoded by *adeRS* to increased expression of the RND-type efflux pump AdeABC and therefore to reduced antimicrobial susceptibility has been described in various studies (19, 21). We have shown previously that *adeB* expression is abolished when *adeRS* is deleted, and we revealed a strain-dependent increased susceptibility to aminoglycosides, carbapenems, fluoroquinolones, glycylcyclines, macrolides, and tetracyclines (23). As the currently available efflux pump inhibitors have no therapeutic value due to their toxicity, more insight to the mechanisms of RND efflux pump regulation is required (24). Targeting the functionality of the two-component system AdeRS seems to be particular promising since the other MDR-associated RND efflux pump AdeIJK is regulated by the repressor AdeN, and affecting it is an inappropriate option to reduce efflux activity.

In the present study, we investigated three amino acid substitutions that were until now uncharacterized, D21V and D26N in AdeR, as well as T156M in AdeS, which were previously identified in clinical A. baumannii isolates which exhibited high tigecycline MICs (17, 18). Since tigecycline is known to be a substrate of RND-type efflux pump AdeABC (25), an increased activity of this pump caused by regulatory mutations was suspected and investigated in the present study.

Various mutations in *adeS* have already been associated with increased AdeABC efflux, including truncation by insertion sequence IS*Aba1*, and several amino acid substitutions (16–18, 26). These mutations were predominantly observed within the histidine kinase, adenylyl cyclase, methyl-accepting protein, and phosphatase (HAMP) and DHp domain (20). Of those, many substitutions were found adjacent or in close proximity to the autophosphorylation site H149, e.g., Q141R, R152K, T153A, T153M, and D167N, indicating a mutational hot spot (17–19, 27). Investigation of AdeS T156M was based on a clinical isolate pair described in a previous study, where the two isolates were identical except for the T156M amino acid substitution in AdeS and susceptibility to tigecycline (17).

Our study confirms that increased *adeB* expression, efflux activity, and, consequently, reduced antimicrobial susceptibility, is triggered by the AdeS T156M amino acid substitution. Since this mutation is located within the same hot spot region of the AdeS kinase domain, although the T156 is not directly involved in the dimerization or *cis*-autophosphorylation of AdeS as revealed by the structure (Fig. S2 in the supplemental material), our findings open up the possibility that these mutations increase the sensitivity of AdeS to environmental stimuli or facilitate activation of the response regulator AdeR by increasing its phosphorylation ratio, which results in increased AdeABC expression (28).

10.1128/msphere.00709-21.3 FIG S2 Crystal structure of AdeS. The residue T156 (red), which is changed to M156 in ABC 153, is located within the dimerization and histidine-containing phosphotransfer domain (DHp) on the same α-helix as the phosphorylation residue H149. Crystal structure obtained from Ouyang et al. (28). Illustration designed with the PyMOL molecular graphics system, version 2.0 (Schrödinger, LLC). Download FIG S2, TIF file, 1.3 MB. Copyright © 2021 Lucaßen et al. 2021 Lucaßen et al. https://creativecommons.org/licenses/by/4.0/ This content is distributed under the terms of the Creative Commons Attribution 4.0 International license.

For detailed characterization, the AdeR with amino acid substitutions D21V and D26N was integrated into the wild-type (WT) *adeRS* backbone of A. baumannii international clone 2 (IC2) reference strain ACICU. Since ACICU is resistant to many commonly used selective markers, it could not be used for characterization of a distinct resistance mechanism. Therefore, *adeRS* of ACICU was cloned into the shuttle vector pJN17/04 and transferred to an *adeRS* deletion strain obtained from ATCC 17978, which is preferred for genetic manipulation because of its well-understood growth dynamics and efficient transformant rates. Although the wild-type ATCC 17978 was shown to be inappropriate for analysis of AdeABC efflux because of low *adeB* expression rates caused by an L172P amino acid substitution in AdeS, it was found to be suitable for characterizing *adeRS* mutations if the WT *adeRS* backbone was replaced with *adeRS* of other reference strains like ACICU (23). Previously, Wen et al. revealed the structure of the response regulator AdeR and showed that residues E19, D20, and K65 and the phosphorylation site D63 form a highly conserved magnesium binding pocket, and they consequently suggested that substitutions of contributing amino acids enhance phosphorylation by AdeS (22). A D20N substitution in AdeR was also shown to increase *adeB* expression and reduce antimicrobial susceptibility (21). Although residue 21 is in close proximity to the binding pocket and the amino acid substitution D21V has been detected in multiple isolates with high tigecycline MICs (17, 18), the contribution of this exchange to increased AdeABC efflux could not be verified in the present study. In contrast to these findings, the AdeR D26N mutation revealed a significant impact on AdeABC expression and subsequently on antimicrobial susceptibility. The residue is located in the α1 helix of AdeR and, consequently, is not directly involved in the dimerization interface (Fig. S3) (22). However, the substitution of aspartic acid to asparagine may alter the binding of AdeR to AdeS during phosphorylation since it is lacking the hydrogen bond acceptor carboxyl group. Nevertheless, the detailed mechanism caused by this mutation remains to be further investigated. Gaining mechanistic insight into the AdeS and AdeR interaction may shed light on the impact of the response regulator D26N substitution on the function of the AdeRS complex.

10.1128/msphere.00709-21.4 FIG S3 Crystal structure of AdeR. The investigated residues D26 (red) and D21 (red) are adjacent to a magnesium binding pocket consisting of residues E19 and D20 and the phosphorylation site D63 and K65. Crystal structure obtained from Wen et al. (22). Illustration designed with the PyMOL molecular graphics system, version 2.0 (Schrödinger, LLC). Download FIG S3, TIF file, 1.4 MB. Copyright © 2021 Lucaßen et al. 2021 Lucaßen et al. https://creativecommons.org/licenses/by/4.0/ This content is distributed under the terms of the Creative Commons Attribution 4.0 International license.

In summary, RND-type efflux pumps are an essential feature for A. baumannii to survive in harsh environments such as a modern intensive care unit by reducing its susceptibility to antimicrobials and biocides. The present study demonstrates the contribution of the AdeS T156M and AdeR D26N substitutions in causing overexpression of the AdeABC efflux pump, resulting in reduced antimicrobial susceptibility. Furthermore, the present data highlight that RND efflux regulators represent a promising target that should be considered in the development of novel antibacterial therapies.

## MATERIALS AND METHODS

### Strains and isolates used in the present study.

The bacterial strains and plasmids used in this study are listed in Table 2. Generation of ATCC 17978 Δ*adeRS* was described previously (23). The clinical isolates ABC153 and ABC154 are an isolate pair, genetically identical apart from a point mutation in *adeS*, causing the amino acid substitution T156M (17).

**TABLE 2:** Strains and plasmids used in this study

Strain or plasmid	Relevant characteristic	Source or reference
E. coli strains		
HST08 Stellar	Chemically competent	TaKaRa Clontech
NEB 5-alpha competent cells	Chemically competent	New England BioLabs
A. baumannii strains		
ACICU	A. baumannii reference strain	30
ABC153	Genetically identical to ABC154 apart AdeS T156M substitution	17
ABC154	Genetically identical to ABC153 apart AdeS wild-type configuration	17
ATCC 17978 Δ*adeRS*	A. baumannii reference strain, *adeRS* deleted	23
17978 *adeR*-wt	ATCC 17978 Δ*adeRS* pJN17/04::*adeRS*^ACICU^	This study
17978 *adeR*-D21V	ATCC 17978 Δ*adeRS* pJN17/04::*adeR*(D21V)*S*^ACICU^	This study
17978 *adeR*-D26N	ATCC 17978 Δ*adeRS* pJN17/04::*adeR*(D26N)*S*^ACICU^	This study
17978 *adeR*-D21V+D26N	ATCC 17978 Δ*adeRS* pJN17/04::*adeR*(D21V,D26N)*S*^ACICU^	This study
Plasmids		
pJN17/04	Shuttle vector	21
pJN17/04::*adeRS*^ACICU^	*adeRS* of ACICU fused to pJN17/04 backbone	This study
pJN17/04::*adeR*(D21V)*S*^ACICU^	AdeR D21V substitution in pJN17/04::*adeRS*^ACICU^	This study
pJN17/04::*adeR*(D26N)*S*^ACICU^	AdeR D26N substitution in pJN17/04::*adeRS*^ACICU^	This study
pJN17/04::*adeR*(D21V,D26N)*S*^ACICU^	AdeR D21V and D26N double substitution in pJN17/04::*adeRS*^ACICU^	This study

### Bacterial growth.

Cells were grown at 37°C in Luria-Bertani (LB) broth or agar. Strains transformed with pJN17/04 were selected by media supplemented with 10 mg/liter kanamycin (21).

### Plasmid purification.

All plasmids used in this study were extracted using the QIAprep Spin miniprep kit (Qiagen, Hilden, Germany) according to the manufacturer’s instructions.

### Transformation of A. baumannii ATCC 17978 Δ*adeRS*.

ATCC 17978 Δ*adeRS* was transformed with purified plasmids by electroporation as described previously (29), using the Gene Pulser II system (Bio-Rad, Munich, Germany). Selection of transformants was performed by growth on LB agar supplemented with 10 mg/liter kanamycin.

### Generation of the shuttle vector pJN17/04::*adeRS*^ACICU^.

The *adeRS* backbone of the reference strain ACICU (30) was used since it represents the wild type of the globally spread A. baumannii international clone 2 (IC2) and was therefore more appropriate for characterization of amino acid substitutions identified in recent clinical isolates than the uncommon *adeRS* configuration of ATCC 17978 (23). Genomic DNA was extracted from a heat-induced crude cell lysate. Amplification of the target DNA was performed using Q5 high-fidelity DNA polymerase (New England BioLabs, Frankfurt, Germany). PCR settings were adjusted according to the PCR product size and the nucleotide sequence of the primer pair O47-O48 (Table S1 in the supplemental material). PCR products were purified using the QIAquick PCR purification kit (Qiagen). The shuttle vector pJN17/04 (21) was linearized by the restriction enzyme ScaI-HF (New England BioLabs). The In-Fusion HD cloning kit (TaKaRa Clontech, Saint-Germain-en-Laye, France) was used for directional cloning of *adeRS* into the shuttle vector. Cloned plasmids were transferred into chemically competent Escherichia coli HST08 cells via heat shock according to the manufacturer’s instructions.

10.1128/msphere.00709-21.1 TABLE S1 Primers used in this study. Download Table S1, DOCX file, 0.01 MB. Copyright © 2021 Lucaßen et al. 2021 Lucaßen et al. https://creativecommons.org/licenses/by/4.0/ This content is distributed under the terms of the Creative Commons Attribution 4.0 International license.

### Introduction of amino acid substitutions into pJN17/04::*adeRS*^ACICU^.

The Q5 site-directed mutagenesis kit (New England BioLabs) was used to exchange single nucleotides within the sequence of *adeR* of pJN17/04::*adeRS*^ACICU^ to introduce the amino acid substitutions D21V and D26N. Primers for PCR amplification of the plasmid, including the corresponding nucleotide exchange, were designed using the online tool NEBaseChanger (New England BioLabs) (Table S1). Modified plasmids were transferred to competent NEB 5-alpha cells by heat shock. Sanger sequencing (LGC Genomics GmbH, Berlin, Germany) was used to confirm the correct nucleotide exchange.

### Antimicrobial susceptibility testing.

Susceptibility to tetracycline, gentamicin (Sigma-Aldrich, Steinheim, Germany), meropenem, amikacin, minocycline, rifampin (Molekula, Newcastle upon Tyne, UK), levofloxacin (Sanofi Aventis, Frankfurt, Germany), ciprofloxacin (Bayer Pharma AG, Berlin, Germany), azithromycin (Pfizer Pharma GmbH, Münster, Germany), chloramphenicol (Serva, Heidelberg, Germany), and erythromycin (AppliChem, Darmstadt, Germany) was tested by agar dilution following current CLSI guidelines (31).

The MIC for tigecycline (Molekula, Newcastle upon Tyne, UK) was determined by broth microdilution following CLSI guidelines (31).

### qRT-PCR.

Expression of *adeB* was evaluated by qRT-PCR as described previously (32). *rpoB* was used as a reference gene, and its expression was quantified concurrently with *adeB* expression. Primers used for the amplification of *adeB* and *rpoB* are listed in Table S1. Freshly prepared RNA (RNeasy; Qiagen) and cDNA (Quantiscript reverse transcriptase; Qiagen) were used to perform qRT-PCR with SYBR green master mix (Qiagen) in triplicates, and the experiment was repeated independently at least three times. The number of *adeB* transcripts in ATCC 17978 Δ*adeRS* pJN17/04::*adeRS*^ACICU^ (here referred to as 17978 *adeR*-wt), pJN17/04::*adeR*(D26N)*S*^ACICU^ (here referred to as 17978 *adeR*-D26N), pJN17/04::*adeR*(D21V)*S*^ACICU^ (here referred to as 17978 *adeR*-D21V), and pJN17/04::*adeR*(D21V, D26N)*S*^ACICU^ (here referred to as 17978 *adeR*-D21V+D26N) transformants were compared with each other. In a separate experiment, the number of *adeB* transcripts was compared between isolate ABC153 and ABC154.

Statistical analysis was performed via GraphPad Prism 9.2.0 (San Diego, California USA) with an unpaired *t* test using the recorded absolute values.

### Accumulation studies.

The AdeABC substrate ethidium bromide was used to investigate efflux activity. Cells grown in LB broth to log phase were washed twice in potassium phosphate buffer (50 mM potassium phosphate buffer, 1 mM MgSO_4_ [pH 7.4]), and adjusted to an optical density at 600 nm of 20. Cells were kept on ice during washing. Pelleting was done in a centrifuge at 4°C and 4,000 × *g* for 5 min. Afterward, the suspension was pipetted to a 96-well Nunclon Delta surface plate (Thermo Fisher Scientific, Schwerte, Germany) and supplemented with 0.2% (wt/vol) glucose (Sigma-Aldrich) and a final ethidium bromide (Merck, Darmstadt, Germany) concentration of 10 μM. The fluorescence was measured in an Infinite M1000 Pro plate reader (Tecan, Crailsheim, Germany) every 15 s over a period of 30 min. The plate reader was set to an excitation wavelength of 530 nm and an emission wavelength of 600 nm.

Accumulation studies were carried out with and without the proton motive force uncoupler carbonyl cyanide *m*-chlorophenylhydrazone (CCCP) (Sigma-Aldrich), used at a final concentration of 500 μM.
