# Supplementary figures and images for: Characterization of Amino Acid Substitutions in the Two-Component Regulatory System AdeRS Identified in Multidrug-Resistant Acinetobacter baumannii
Source: mSphere. 2021 Nov 24;6(6):e00709-21. doi: 10.1128/msphere.00709-21 (PMC8612257; doi:10.1128/msphere.00709-21)

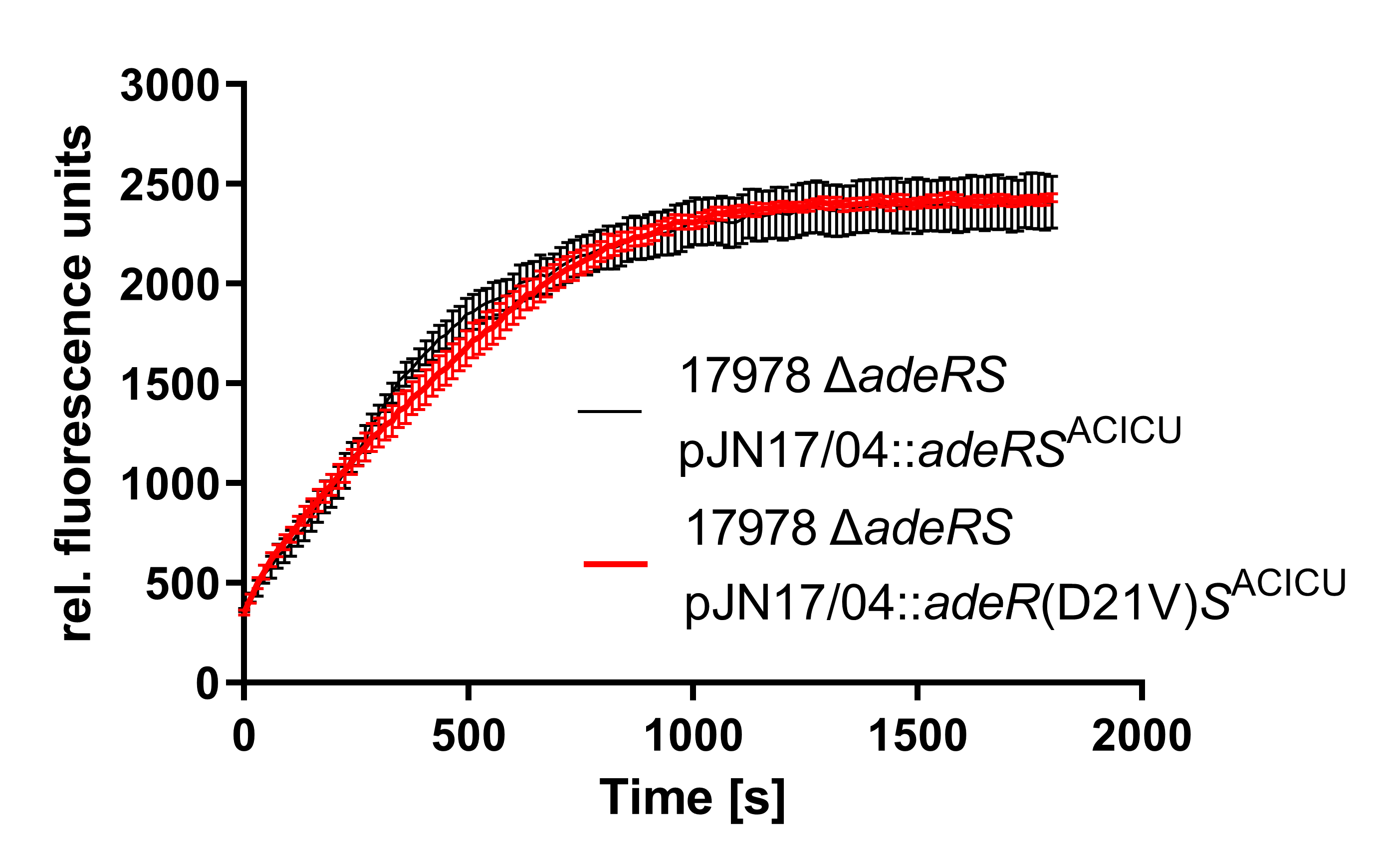

Supplement: FIG S1 [file msphere.00709-21-sf001.tif]

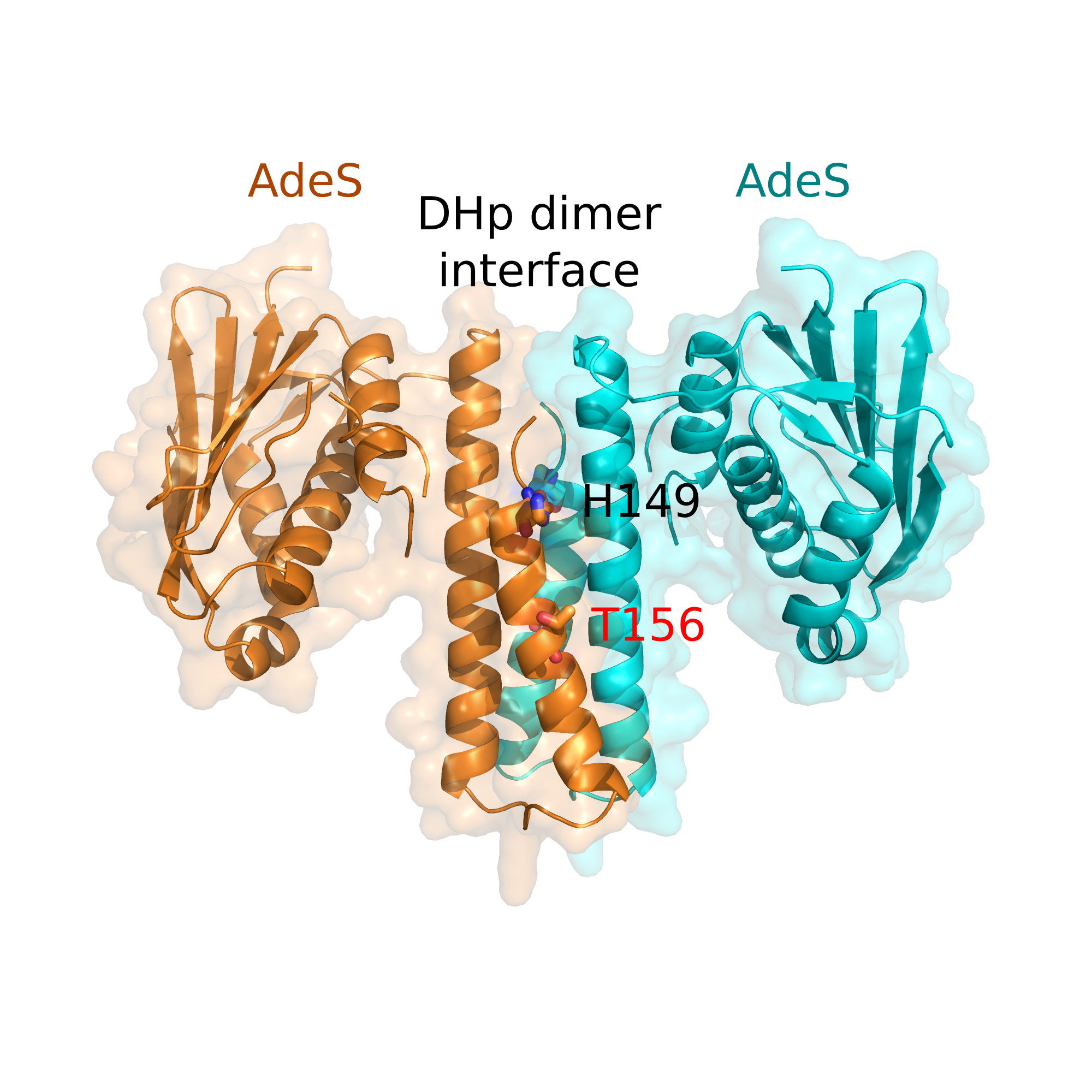

Supplement: FIG S2 [file msphere.00709-21-sf002.tif]

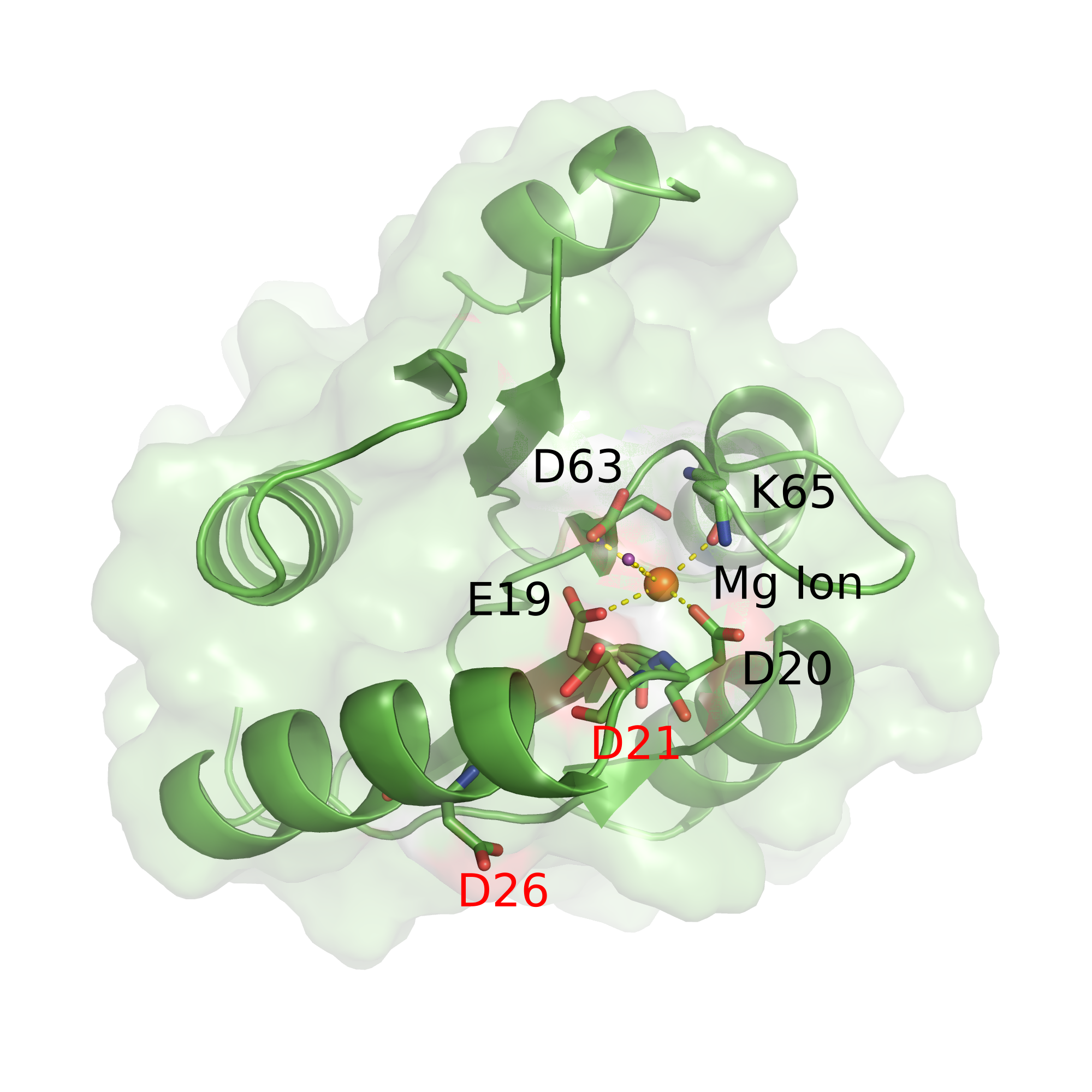

Supplement: FIG S3 [file msphere.00709-21-sf003.tif]
