# Supplementary material for: Characterization of Amino Acid Substitutions in the Two-Component Regulatory System AdeRS Identified in Multidrug-Resistant Acinetobacter baumannii
Source: mSphere. 2021 Nov 24;6(6):e00709-21. doi: 10.1128/msphere.00709-21 (PMC8612257; doi:10.1128/msphere.00709-21)
Supplement: TABLE S1 [file msphere.00709-21-st001.docx]

**Supplemental Table S1:** Primers used in this study.

| **Target** | **Primer name** | **Sequence (5′ - 3′)** | **Size (bp)** | **Experiment** |
| --- | --- | --- | --- | --- |
| *adeRS*^ACICU^ | O47 | gccactcatcgcagtATTGCGGTTGAATGCTTAATACAC | 2,149 | InFusion cloning into pJN17/04 |
|  | O48 | atgaattacaacagtAGACAGCTTGGGATCAGGAAGTC |  |  |
| pJN17/04::*adeRS* | N72 | CGATATTGGC**A**ACATTATTGAAAATTATTTAAAAC | 8,473 | Site-directed mutagenesis *adeR*(D26N) |
|  | N73 | TAGTCATCTTCTACCACAAG |  |  |
| pJN17/04::*adeRS* | O27 | GTAGAAGATG**T**CTACGATATTGG | 8,437 | Site-directed mutagenesis *adeR*(D21V) |
|  | O28 | CACAAGAATAACTTTATCTTGG |  |  |
| *adeR* | D31 | GGAGTAAGTGTGGAGAAATACGG | 937 | Sanger sequencing |
|  | K57 | ACCCAGTACTACAGAAAATAGCG |  |  |
| *adeS* | K28 | AAACTTGCTCAATACGACGG | 518 | Sanger sequencing |
|  | K42 | AAAGCGTTTTATTGTGCCAA |  |  |
| *rpoB* | C65 | GAGTCTAATGGCGGTGGTTC | 110 | qRT-PCR |
|  | C66 | ATTGCTTCATCTGCTGGTTG |  |  |
| *adeB* | A48 | GTATGAATTGATGCTGC | 992 | Standard for qRT-PCR |
|  | A49 | CACTCGTAGCCAATACC |  |  |
|  | M26 | CAAGGACGTATGCAACAAGT | 103 | qRT-PCR |
|  | M27 | CTAATTGACCGCTTGAACCC |  |  |

Lowercase: tails for InFusion cloning

Bold and in a box: site of nucleotide substitution
